# Real‐time evaluation of the biocompatibility of calcium silicate‐based endodontic cements: An in vitro study

**DOI:** 10.1002/cre2.714

**Published:** 2023-03-02

**Authors:** Soledad Rebolledo, Raúl Alcántara‐Dufeu, Luis Luengo Machuca, Luciano Ferrada, Gabriela Alejandra Sánchez‐Sanhueza

**Affiliations:** ^1^ Departamento de Odontología Restauradora, Facultad de Odontología Universidad de Concepción Concepción Chile; ^2^ Departamento de Salud Pública, Facultad de Odontología Universidad de Concepción Concepción Chile; ^3^ Facultad de Ciencias Biológicas Universidad de Concepción Concepción Chile

**Keywords:** biocompatibility, cell proliferation, human periodontal ligament cells, IncuCyte S3

## Abstract

**Introduction:**

An ideal filling material should hermetically seal the communication pathways between the canal system and surrounding tissues. Therefore, during the last few years, the development of obturation materials and techniques to create optimal conditions for the proper healing of apical tissues has been a focus of interest. The effects of calcium silicate‐based cements (CSCs) on periodontal ligament cells have been investigated, and promising results have been obtained. To date, there are no reports in the literature that have evaluated the biocompatibility of CSCs using a real‐time live cell system. Therefore, this study aimed to evaluate the real‐time biocompatibility of CSCs with human periodontal ligament cells (hPDLCs).

**Methodology:**

hPDLC were cultured with testing media of endodontic cements for 5 days: TotalFill‐BC Sealer, BioRoot RCS, Tubli‐Seal, AH Plus, MTA ProRoot, Biodentine, and TotalFill‐BC RRM Fast Set Putty. Cell proliferation, viability, and morphology were quantified using real‐time live cell microscopy with the IncuCyte S3 system. Data were analyzed using the one‐way repeated measures (RM) analysis of variance multiple comparison test (*p* < .05).

**Results:**

Compared to the control group, cell proliferation in the presence of all cements was significantly affected at 24 h (*p* < .05). ProRoot MTA and Biodentine lead to an increase in cell proliferation; there were no significant differences with the control group at 120 h. In contrast, Tubli‐Seal and TotalFill‐BC Sealer inhibited cell growth in real‐time and significantly increased cell death compared to all groups. hPDLC co‐cultured with sealer and repair cements showed a spindle‐shaped morphology except with cements Tubli‐Seal and TotalFill‐BC Sealer where smaller and rounder cells were obtained.

**Conclusions:**

The biocompatibility of the endodontic repair cements performed better than the sealer cements, highlighting the cell proliferation of the ProRoot MTA and Biodentine in real‐time. However, the calcium silicate‐based TotalFill‐BC Sealer presented a high percentage of cell death throughout the experiment similar to that obtained.

## 1 INTRODUCTION

In addition to the traditional concepts of debridement, disinfection, and adequate root filling to seal the possible communications with periodontium, the success of endodontic treatment depends on multiple factors (Ng et al., 2008). However, most failures occur as a result of bacterial persistence and leakage of irritants into the periodontal tissues (Siqueira & Rôças, 2008). Therefore, an ideal filling material should hermetically seal the communication pathways between the canal system and surrounding tissues.

It has been reported that the periodontal ligament is essential for the regeneration of periodontal tissues as it contains a heterogeneous population of cells (Mu et al., 2017). These cells have a high potential for self‐renewal and pluripotency; therefore, they can serve as seed cells for bone regeneration (Seo et al., 2004).

If the filling material, especially endodontic cement, comes into direct contact with the periradicular tissues for prolonged periods, it can lead to irritation and delay tissue healing (Jung et al., 2019). Therefore, biocompatibility is defined as the ability of a material to achieve an adequate and advantageous host response in specific applications. This is an essential requirement for all filling materials (Al‐Haddad & Che Ab Aziz, 2016). However, types of cement tend to show a certain degree of toxicity, especially freshly mixed cement, which then tends to decrease with setting (Fonseca et al., 2019). Several cytotoxicity assays involve the measurement of cell membrane integrity and metabolic activity to assess cell morphology and viability (Riss et al., 2004). While these assays provide important information on the biocompatibility of materials, they have several limitations, such as dependence on proliferation, confluency, cell morphology, and the metabolic state of the cells. In addition, another limitation of great relevance is that these assays are conditioned to be “end point” assays, that do not allow quantification in time and are limited to an arbitrarily determined time point (Armenta & Dixon, 2020). Additionally, the only definition of cell death is loss of plasma membrane integrity (Galluzzi et al., 2018). Thus, assays that measure metabolic activity do not allow the differentiation of living cells from dead ones, which can lead to false positive/negative results while studying the cytopathic effects of compounds to be tested. This is because, despite cell death, there can be a decrease in metabolism. Although these limitations are known, to date, there are no reports in the literature that have evaluated the biocompatibility of calcium silicate‐based cements (CSCs) using the state‐of‐the‐art IncuCyte®S3 real‐time live cell system.

CSCs were introduced in modern dentistry more than two decades ago with diverse applications (Torabinejad et al., 2018). Owing to their excellent biocompatibility, bioactivity, and sealing ability, they are used in multiple clinical applications, including pulp regeneration and hard tissue repair (Parirokh et al., 2018). During the last few years, the effects of CSCs on periodontal ligament cells have been investigated, and promising results have been obtained with regard to assessing cell viability, migration, morphology, and adhesion, in addition to their bioactivity and mineralization capacity (S. W. Chang et al., 2014; Lee et al., 2019; López‐García et al., 2020; T. Luo et al., 2018). However, there are significant variabilities in the reported findings depending on the methodology and time frame in which the studies were undertaken.

Thus, it becomes necessary to study the real‐time interactions of periodontal ligament cells in contact with these materials. This would permit the observation of the biological effects and enable tracking throughout the incubation period and not restricted to a particular timeIncuCyte® S3 technology, according to the manufacturer, uses real‐time monitoring to assess cell health and viability, with the advantage that cells are not destroyed during the experimental process and can be further characterized using other technologies (https://www.sartorius.com/en/applications/life-science-research/cell-analysis/live-cell-assays/cell-health-proliferation). Therefore, this study aimed to evaluate the real‐time biocompatibility of CSCs with human periodontal ligament cells (hPDLC). The null hypotheses tested were that CSCs do not show better biocompatibility on periodontal ligament cells than those cements commonly used in endodontic treatment.

## 2 MATERIALS AND METHODS

**Figure 1:**
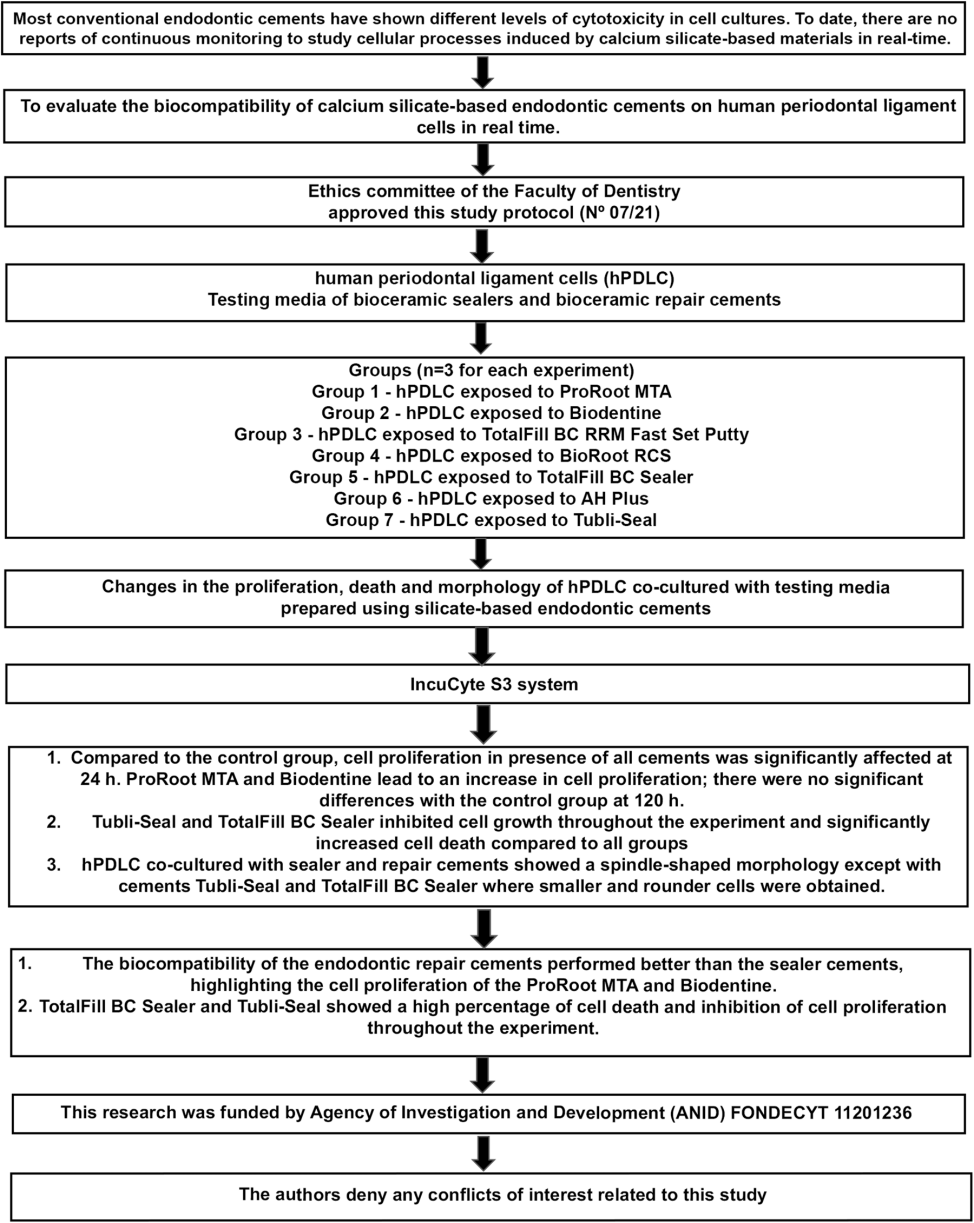
The study design according to the Preferred Reporting Items for Laboratory studies in Endodontology (PRILE), 2021 guidelines.

For this in vitro study, healthy impacted third molars extracted from patients between 18 and 25 years were used. Prior authorization of the patient by means of informed consent included in the protocol approved by the Scientific Ethics Committee C.E.C.No.07/21 was obtained before performing the exodontia and subsequently filed. The steps in this study are depicted in the PRILE flowchart (Figure 1).

### 2.1 Materials and reagents

The following types of cement were used for the study:

Sealer cements: BioRoot™ RCS (Septodont) and TotalFill® BC Sealer™ (FKG), Tubli‐Seal™ (Kerr), and AH Plus® (Dentsply).

Repair cements: TotalFill® BC RRM™ Fast‐Set Putty (FKG) and Biodentine® (Septodont) and ProRoot® MTA (Dentsply).

### 2.2 Cell culture

hPDLC were cultured according to previously described protocols, with minor modifications (Y. Wang, Du, et al., 2020). Briefly, the teeth were washed three times with PBS to remove blood debris, and the periodontal ligament was separated from the middle third of the root within the first few hours of a tooth extraction with a sterile scalpel. The tissue was crushed into small pieces and incubated with trypsin for 30 min to promote cell disintegration. Subsequently, the tissue was resuspended in a complete medium and mechanically homogenized. Excess tissue pieces were allowed to settle, and the supernatant was seeded onto 100 mm plates for establishing the culture. Cells were cultured for 30 days in minimal essential medium (MEM; HyClone) supplemented with 5% fetal bovine serum (HyClone), GlutaMax (Gibco, Life Technologies), 100 IU/mL penicillin, 100 μg/mL streptomycin (Gibco, Life Technologies), and 0.25 μg/mL amphotericin B (Gibco, Life Technologies) and incubated at 37°C with 5% CO2 in a humidity‐controlled incubator.

### 2.3 Preparation of cements

All materials were mixed under sterile conditions according to the manufacturer's instructions. Three 4 × 3 mm disks of each cement were made with amalgam holders and allowed to stand for 24 h at 37°C and 100% humidity. The samples were placed in 24‐well culture plates, washed twice with buffer solution, and dried in laminar air flow for 24 h at room temperature. They were then UV‐sterilized for 15 min each side before being added to the cell culture for indirect contact assays.

### 2.4 Biocompatibility evaluation

The three disks of each cement were placed in Eppendorf tubes, and 1 mL of supplemented MEM was added to the disks and incubated for 3 days at 37°C. This conditioned liquid was used as the test medium for the assays. Following centrifugation at 3500 rpm for 10 min, the liquid was passed through a 0.22 µm size membrane filter and added to the cell cultures in the same 96‐well plate. Tubli‐Seal™ (Kerr) and AH Plus® were used as a positive control. Unexposed periodontal ligament cells used as negative control were maintained under the same conditions. Cell proliferation, viability, and morphology were quantified using real‐time live cell microscopy with the IncuCyte®S3 Live‐Cell Analysis System (Essen BioScience, Sartorius; Cat. no.: 9500‐4647‐F00) under a 10x objective. Data were recorded every 3 h once the culture was initiated, until Day 5, and analyzed using IncuCyte® Cell‐by‐Cell Analysis Software (Essen BioScience, Sartorius; Cat. no.:9600‐0031).

### 2.5 Statistical analysis

An analysis of variance test with multiple comparisons (Tukey's method) was used, with 95% confidence intervals for calculating the differences. Differences were considered statistically significant at the 5% level. Data were analyzed using GraphPad Prism 6.0 (GraphPad Inc.).

## 3 RESULTS

### 3.1 Cell proliferation

**Figure 2:**
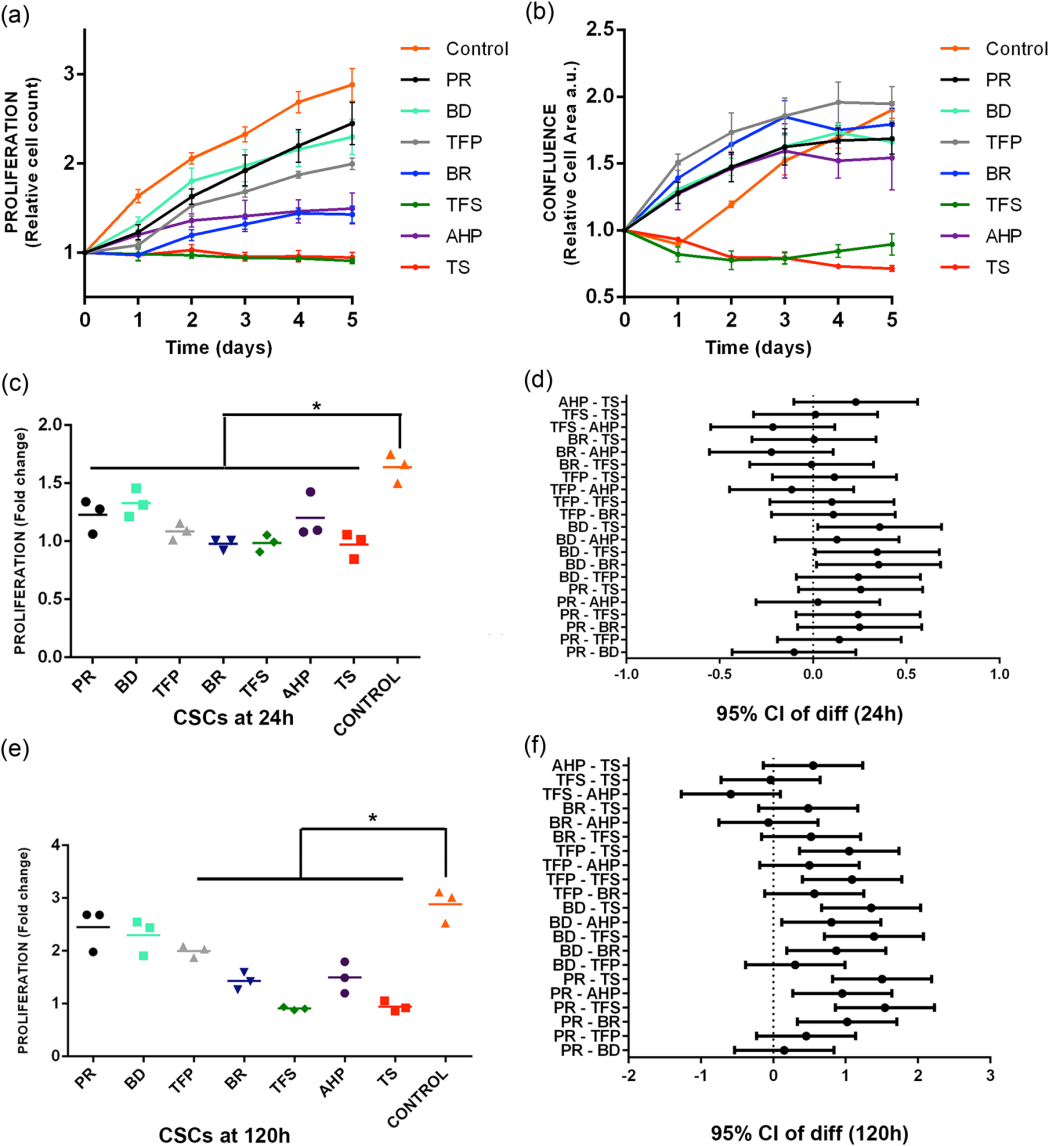
The effects of the materials on human periodontal ligament cells (hPDLCs) proliferation. (a) Real‐time behavior of hPDLSC proliferation exposed to the cement extracts throughout the experiment. (b) Real‐time behavior of hPDLSC confluency exposed to the cement extracts throughout the experiment. (c) Comparison of cell proliferation with the control group at 1 day. (d) Comparison of cell proliferation between cements at 1 day. (e) Comparison of cell proliferation with the control group at 5 days. (f) Comparison of cell proliferation between cements at 5 days. AHP, AH Plus; BD, Biodentine; BR, BioRoot RCS; PR, ProRoot MTA; TFP, TotalFill BC RRM Fast Set Putty; TFS, TotalFill BC Sealer; TS, Tubli‐Seal. *Significant difference when compared with de control group (*p* ˂ .05).

**Figure 3:**
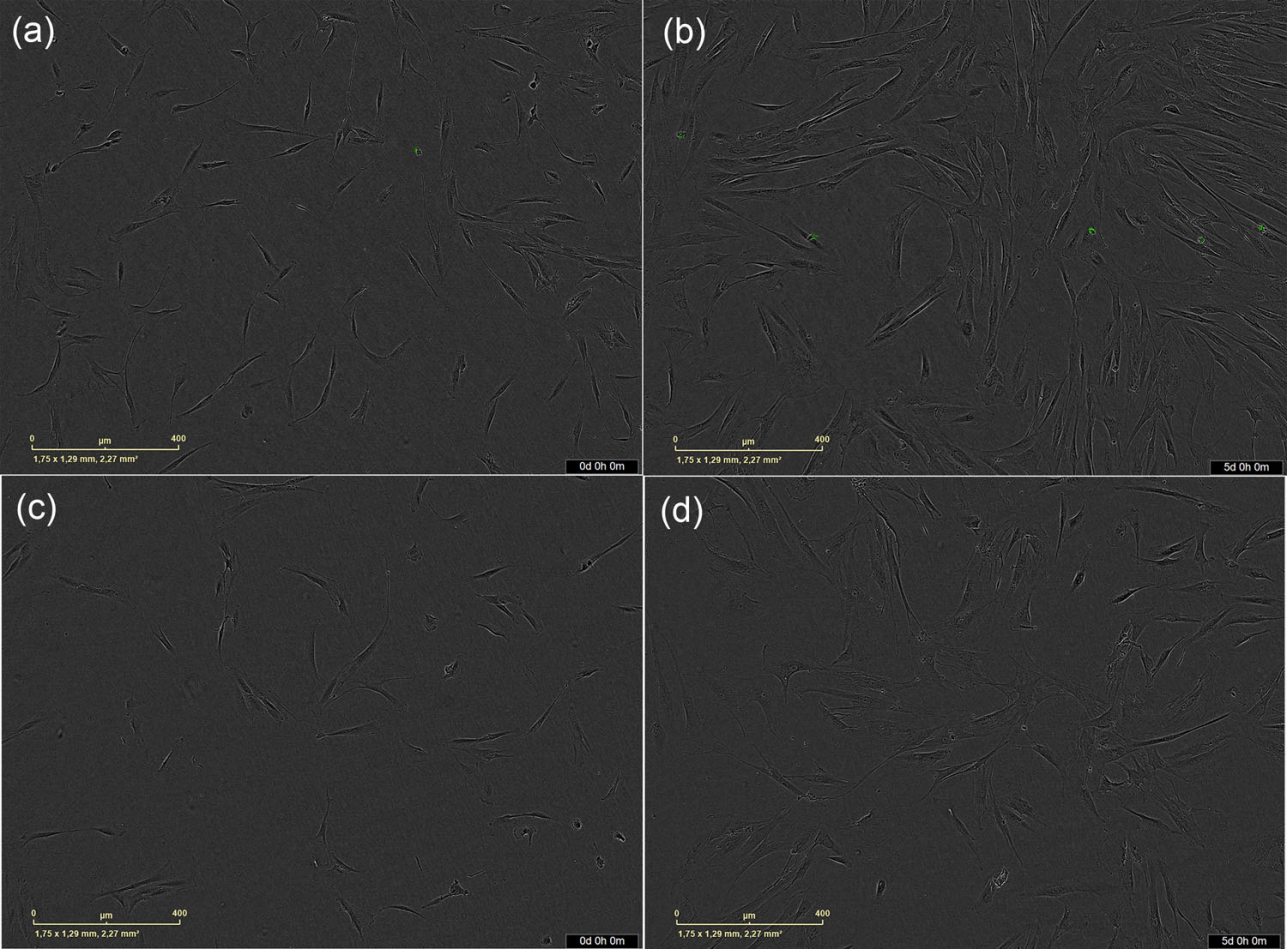
Human periodontal ligament cells cultured in (a) Biodentine test medium at 0 h. (b) Biodentine test medium at 5 days. (c) ProRoot MTA test medium at 0 h. (d) ProRoot MTA test medium at 5 days. IncuCyte®S3 Live‐Cell Analysis System under a 10x objective.

The cells in the control group reached a higher proliferation, with a tripling of the population being observed on Day 5 (final time), followed by the ProRoot MTA, Biodentine, TotalFill BC RRM Fast Set Putty, AH Plus, and BioRoot RCS tests. In contrast, inhibition of cell proliferation was noted in Tubli‐Seal and TotalFill BC Sealer (Figure 2a). It is worthwhile mentioning that these cements caused a decrease in the space occupied by the cells within the well, resulting in significant differences in cell confluency compared to the control group (Figure 2b). At 1 day, cells treated with all cements had significantly lower cell proliferation than the cells of the control group implying that cell proliferation was affected by the presence of cements in all groups during the first day (Figure 2c). Only cells treated with Biodentine had a significantly better proliferation than BioRoot RCS, TotalFill Sealer, and Tubli‐Seal in this period (Figure 2d). This effect changed throughout the real‐time, showing at 5 days that only ProRoot MTA and Biodentine caused no significant changes in cell proliferation compared with the control group (Figure 2e). When the cements were compared, the Biodentine, ProRoot MTA, and TotalFill Fast Set Putty showed significant differences in cellular proliferation, especially in comparison with the Tubli‐Seal and TotalFill BC Sealer in the indicated period (Figure 2f). hPDLSC cultured in Biodentine and MTA ProRoot test media at 0 and 5 days are shown in Figure 3.

### 3.2 Cell viability

**Figure 4:**
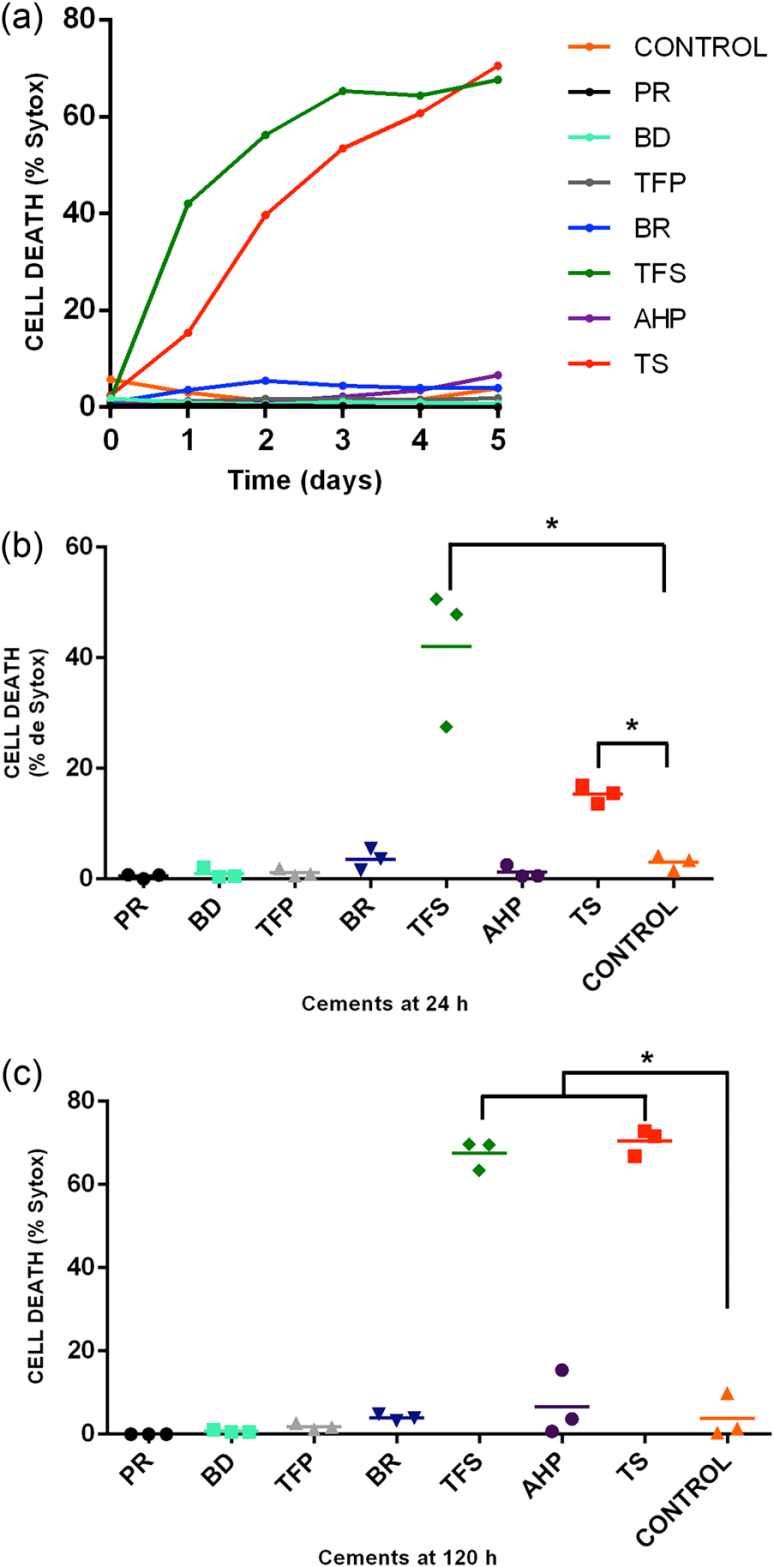
The effects of the materials on human periodontal ligament cell (hPDLC) viability. (a) Real‐time behavior of hPDLSC viability exposed to the cement extracts throughout the experiment (b) Analysis at 1 day of cell death. (c) Analysis at 5 days of cell death. AHP, AH Plus; BD, Biodentine; BR, BioRoot RCS; PR, ProRoot MTA; TFP, TotalFill BC RRM Fast Set Putty; TFS, TotalFill BC Sealer; TS, Tubli‐Seal. *Significant difference when compared with de control group (*p* ˂ .05).

**Figure 5:**
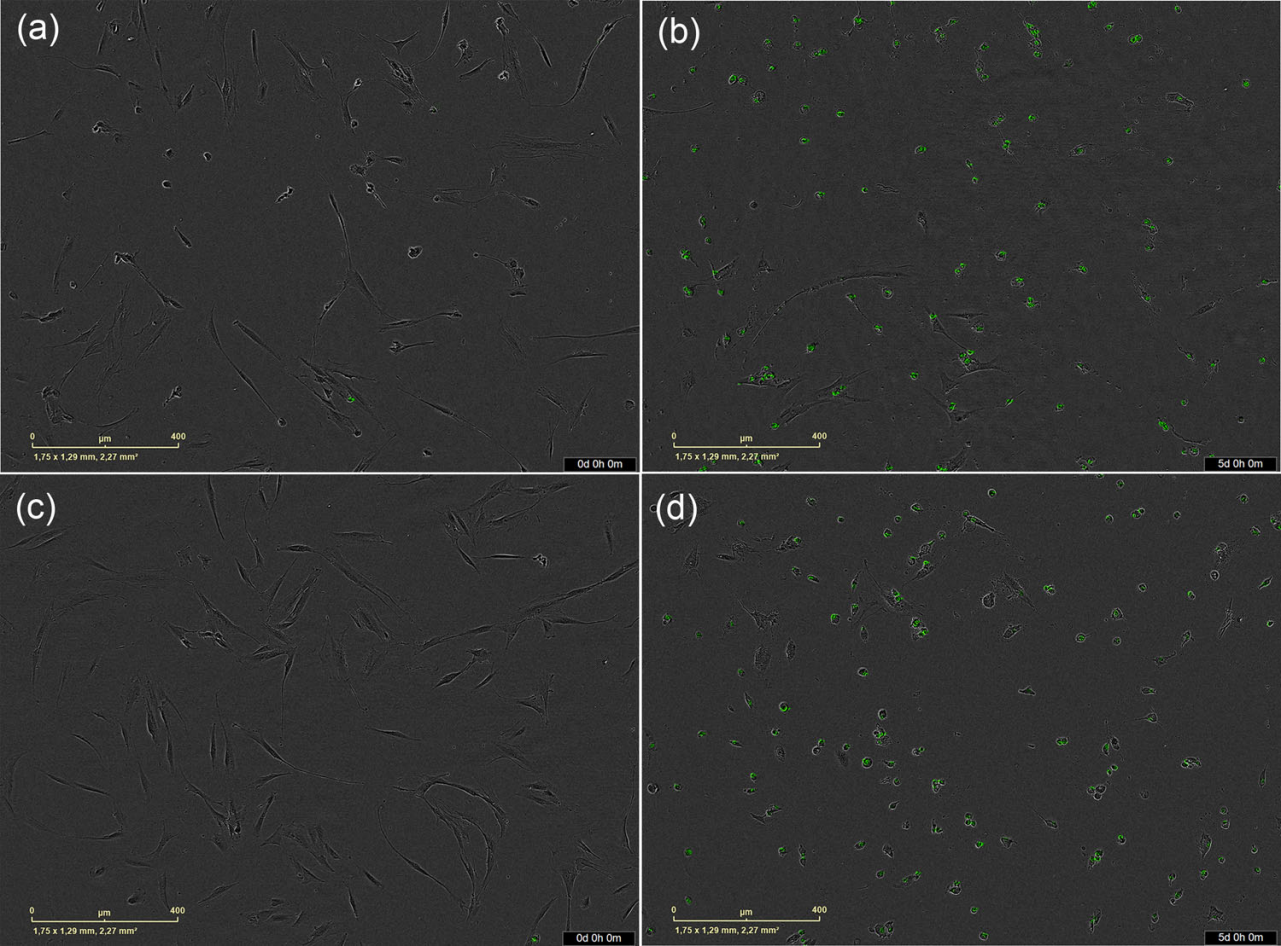
Human periodontal ligament cells cultured in (a) TotalFill BC Sealer test medium at 0 h. (b) TotalFill BC Sealer test medium at 5 days. (c) Tubli‐Seal test medium at 0 h. (d) Tubli‐Seal test medium at 5 days. IncuCyte®S3 Live‐Cell Analysis System under a 10x objective.

Tubli‐Seal and TotalFill BC Sealer affected the cell viability of a high percentage of cells compared with the control from the first day to the end of the experiment. The other cements did not cause cell death during the experimental period (Figure 4a). This difference in the percentage of dead cells was significant in both the cements groups compared to the control group (Figure 4b) and for the rest of the cements; significance was obtained both at 24 h as well as at the end of the experiment (Figure 4c). The percentage of dead cells was higher in TotalFill BC Sealer than in Tubli‐Seal at 24 h; however, this relationship changed at 5 days, with the percent viability being similar in the two. hPDLSC cultured in Tubli‐Seal and TotalFill BC Sealer test media at 0 and 5 days are shown in Figure 5.

### 3.3 Cell morphology

**Figure 6:**
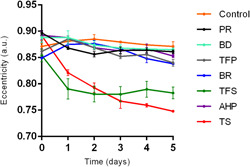
Morphology of human periodontal ligament cells. Tubli‐Seal and TotalFill Sealer cements altered cell morphology; more rounded cells were seen (values are close to 0).

hPDLC exhibited spindle‐shaped morphology, characteristic of fibroblasts when cultured in the in vitro cell system. This morphology was maintained during the test period with the control group and test media of all cements, except Tubli‐Seal and TotalFill Sealer. As incubation time increased, this characteristic shape changed, and smaller and rounded cells were seen (Figure 6). This shape was predominantly associated with cell death, and coincided with the higher percentages of dead cells in test media of these two cements and reduced cell confluency; since being smaller, they occupied less space within the well.

Supporting Information: Video files of hPDLC cultured with CSCs in real‐time, available online.

## 4 DISCUSSION

The hPDLC and their interaction with cements in periapical tissues can modulate the inflammatory response, its resolution, and initiation of lost tissue repair (Giraud et al., 2018). The present study used cell cultures obtained from freshly extracted third molars to simulate clinical conditions. The biocompatibility of different endodontic cements was evaluated using three variables: cell proliferation, viability, and morphology, by real‐time live cell monitoring with the IncuCyte®S3 microscopy system. Unlike most studies that evaluate the cytotoxicity of cements with assays that measure the metabolic activity of cells (Camps et al., 2015; S. W. Chang et al., 2014; Fonseca et al., 2019; López‐García et al., 2020), the present study used a state‐of‐the‐art method (S. Wang, Huang, et al., 2020). This permitted the evaluation and monitoring of these variables in real‐time and not at an endpoint, without altering the cell cultures and depending on other variables such as the cell metabolic activity.

The results of the first day allowed us to observe a significant difference in cell proliferation between the control group and the rest of the cement groups. However, by the end of the assay, no differences in cell proliferation were found between the MTA ProRoot and Biodentine compared to the control group. The desirable results obtained with these two cements are corroborated by several systematic reviews that demonstrated the high biocompatibility of these materials, in addition to other properties, such as bioactivity, sealing capacity, adhesion, solubility, and antimicrobial efficacy (Escobar‐García et al., 2016; Parirokh et al., 2018). Previous studies have shown that Biodentine exhibits equally good biocompatibility with periodontal ligament cells compared to MTA (Jung et al., 2014; Tang et al., 2019). These results for Biodentine, are consistent with previous studies wherein MTT assays showed similar proliferative activity and cytotoxicity in different cell lines (Abuarqoub et al., 2020; Attik et al., 2014). In contrast, some studies have also reported decreased cell viability when exposed to high concentrations of Biodentine (Hasweh et al., 2018; Z. Luo et al., 2014). It should be noted that the cell morphology remained spindle‐shaped throughout the present study in both cements, which is consistent with previous reports (T. Luo et al., 2018; Lv et al., 2017).

MTA is the most widely studied cement and is considered as the “gold standard” in the investigations of calcium silicate‐based materials. Unlike MTA, only a few studies in vivo have evaluated the biocompatibility of other CSCs such as Biodentine and Bioaggregates (Song et al., 2021). In addition, the results of various in vitro models are variable owing to discrepancies in cell types and sample preparation methods, which makes it difficult to compare the results and derive conclusions across the studies. Hence, it is necessary to establish a standard research model using well‐defined experimental procedures and evaluation criteria (Hosseinpour et al., 2022).

The biocompatibility of BioRoot RCS has been reported in several studies, where it has been shown that BioRoot RCS extracts had dose‐ and time‐dependent effects on cell responses, showing excellent cytocompatibility in terms of cell proliferation, viability, and migration (Collado‐González et al., 2017; Dimitrova‐Nakov et al., 2015; Siboni et al., 2017). In the present study, BioRoot RCS increased the proliferation of PDLCs starting on Day 1; however, during the course of the experiment, it was significantly lower than that of the control group. These findings are consistent with results of previous studies wherein BioRoot RCS showed a significant reduction in viability of PDLC after 2 days of contact with the test medium compared to the control medium; however, from the second day onwards, there was no variation in cell viability in extracts with BioRoot RCS (Camps et al., 2015). It has also been reported that despite obtaining significant differences in cell viability compared to the control group, there was a regeneration of hPDLC over time when in contact with BioRoot RCS, and lower cytotoxicity was noted, indicating that BioRoot RCS is biocompatible as well as bioactive (Jung et al., 2019).

In this study, the TotalFill BC sealer inhibited cell proliferation throughout the experiment, and a significant increase in cell death was also noted. This is contrary to a previous study where TotalFill BC Sealer was shown to have no effect on the viability of hPDL stem cells (hPDLSCs) relative to the control group during the first 24 h, though the cell viability decreased at 48 and 72 h. AH plus cement showed significantly lower cytotoxicity than TotalFill BC Sealer throughout the experiment (López‐García et al., 2019). It has been shown that hPDLSC proliferation levels in the presence of TotalFill BC Sealer extracts were similar to that obtained using a control medium and were also significantly higher than the levels achieved from 48 h of incubation with AH Plus (Rodríguez‐Lozano et al., 2017). The differences in these results may be due to the different methodologies used in the investigations since even though the two studies used different dilutions of the cement extracts, the cell viability was estimated by the MTT assay.

With respect to the Tubli‐Seal zinquenolic cement, varied evidence suggests its high level of toxicity (Komabayashi et al., 2020; Özdemir & Kopac, 2022). Zinc oxide eugenol‐based sealers have been shown to be irritant and cytotoxic agents (Gulati et al., 1991) that activate a complement‐mediated immune response, as well as exhibit significant fibroblast cytotoxicity (Serene et al., 1988). An in vitro evaluation of the influence of the addition of antioxidants (pachymic acid) on the cytotoxic properties of different sealers, including ZOE (Tubli‐Seal), epoxy resin (AH Plus), and calcium hydroxide (Sealapex)‐based sealants, showed that the addition of pachymic acid led to a significant decrease in cytotoxicity (Arun et al., 2017). The morphological changes in cells exposed to Tubli‐Seal extracts observed in the present investigation have also been reported in a previous study (M. C. Chang et al., 2010).

The differences found in the behavior of repair and sealer cements could be related to the physical properties of these materials, especially their solubility. The solubility of a sealer should not be superior to 3% by mass according to the requirements codified in ISO 6876 and ANSI/ADA 57 (Aminoshariae et al., 2022). Previous studies show that most of these sealers meet many criteria for ideal properties, but limitations were found in solubility and dimensional stability after immersion in water compared to standard resin‐based sealers (Eskandari et al., 2022; Jafari & Jafari, 2017).

Nowadays, several cytotoxicity screening methods are used for studying endodontic materials, and it has been shown that various cytotoxicity screening methods yield a spectrum of results for the same material (Hosseinpour et al., 2022). It should be emphasized that any form of in vitro cell culture testing is very different from the clinical setting; therefore, few conclusions can be extrapolated to clinical situations, and only a statistical approximation of the biocompatibility of an endodontic material can be derived from the in vitro data. CD4+ T lymphocytes and CD14+ monocytes, normal diploid cells with mitotic rates, and mitochondrial function are relatively similar in vivo. Their use is suggested to record the effect of biomaterials, even at the DNA level, as the cells can simulate periapical inflammation, characterized by an infiltration of leukocytes, mainly polymorphonuclear cells, followed by monocytes (Velard et al., 2010). Among the limitations of this study, the approximation of the clinical behavior of these materials can be improved with bioactivity and cytokine secretion assays to evaluate their action in inflammation, repair, and healing of tissue lesions (Castro‐Jara et al., 2023; Talabani et al., 2020). Additionally, this study only evaluated biocompatibility indirectly through a test medium, but it should also be evaluated through direct contact of cells with these materials to have more complete results that can serve as a guide for the next clinical work. The results of the present study can be considered a starting point for future research on the biocompatibility of endodontic cements, suggesting the use of techniques using real‐time instead of end time as a standard. To complement these results, in addition to cell counting techniques, it is suggested to evaluate biological responses with cellular metabolic depletion methods and advance to randomized clinical trials.

## 5 CONCLUSIONS

The biocompatibility of the calcium silicate‐based endodontic repair cements was better than that of the sealer cements, with ProRoot MTA and Biodentine showing the most promising results. The TotalFill BC Sealer calcium silicate‐based sealer and Tubli‐Seal showed a higher percentage of cell death and inhibition of cell proliferation throughout the experiment, indicating greater cytotoxic effects than AH Plus, a resin‐based cement commonly used in endodontics. Overall, the results suggest that both cements contribute to the delayed healing and regeneration of periapical lesions. Nevertheless, further in vitro studies with similar methodologies are required to compare these results and derive more conclusive evidence.

## AUTHOR CONTRIBUTIONS

**Soledad Rebolledo**: Term; conceptualization; methodology; software; validation; formal analysis; investigation; resources; data curation; writing – original draft. **Raúl Alcántara‐Dufeu**: Resources; writing – original draft. **Luis Luengo Machuca**: Software; validation; formal analysis; data curation; writing – original draft. **Luciano Ferrada**: Term; conceptualization; methodology; software; validation; formal analysis; investigation. **Gabriela Alejandra Sánchez‐Sanhueza**: Term; conceptualization; methodology; software; validation; formal analysis; investigation; resources; data curation; writing – original draft; writing – review and editing; visualization; supervision; project administration.

## CONFLICTS OF INTEREST STATEMENT

The authors declare no conflicts of interest.

## Supporting information

Supporting information.

Supporting information.

Supporting information.

Supporting information.

## Data Availability

Data sharing is not applicable as no new data were generated.
